# Hospital Utilisation in Indigenous and Non-Indigenous Infants under 12 Months of Age in Western Australia, Prospective Population Based Data Linkage Study

**DOI:** 10.1371/journal.pone.0154171

**Published:** 2016-04-27

**Authors:** Kimberley McAuley, Daniel McAullay, Natalie A. Strobel, Rhonda Marriott, David N. Atkinson, Julia V. Marley, Fiona J. Stanley, Karen M. Edmond

**Affiliations:** 1 School of Paediatrics and Child Health, University of Western Australia, Perth, Western Australia, Australia; 2 Kurongkurl Katitjin, Centre for Indigenous Australian Education and Research, Edith Cowan University, Perth, Western Australia, Australia; 3 School of Psychology and Exercise Science, Murdoch University, Perth, Western Australia, Australia; 4 The Rural Clinical School of Western Australia, The University of Western Australia, Broome, Western Australia, Australia; 5 Telethon Kids Institute, Perth, Western Australia, Australia; Curtin University, AUSTRALIA

## Abstract

**Background:**

Indigenous infants (infants aged under 12 months) have the highest hospital admission and emergency department presentation risks in Australia. However, there have been no recent reports comparing hospital utilisation between Indigenous and non-Indigenous infants.

**Methods:**

Our primary objective was to use a large prospective population-based linked dataset to assess the risk of all-cause hospital admission and emergency department presentation in Indigenous compared to non-Indigenous infants in Western Australia (WA). Secondary objectives were to assess the effect of socio-economic status (Index of Relative Socio-Economic Disadvantage [IRSD]) on hospital utilisation and to understand the causes of hospital utilisation.

**Findings:**

There were 3,382 (5.4%) Indigenous and 59,583 (94.6%) non-Indigenous live births in WA from 1 January 2010 to 31 December 2011. Indigenous infants had a greater risk of hospital admission (adjusted odds ratio [aOR] 1.90, 95% confidence interval [95% CI] 1.77–2.04, p = <0.001) and emergency department presentation (aOR 2.15, 95% CI 1.98–2.33, p = <0.001) compared to non-Indigenous infants. Fifty nine percent (59.0%) of admissions in Indigenous children were classified as preventable compared to 31.2% of admissions in non-Indigenous infants (aOR 2.12, 95% CI 1.88–2.39). The risk of hospital admission in the most disadvantaged (IRSD 1) infants in the total cohort (35.7%) was similar to the risk in the least disadvantaged (IRSD 5) infants (30.6%) (aOR 1.04, 95% CI 0.96–1.13, p = 0.356).

**Interpretation:**

WA Indigenous infants have much higher hospital utilisation than non Indigenous infants. WA health services should prioritise Indigenous infants regardless of their socio economic status or where they live.

## Introduction

Over the last ten years a key element of the Australian Federal Government strategy to ‘Close the gap’[1, 2] in health outcomes between Australian Aboriginal and Torres Strait Islander (hereafter Indigenous) and non-Indigenous infants has been to improve access to urban and remote area health services for Indigenous mothers and children. This has included increased funding for hospitals[3], specialist outreach services[4], and care coordination[5–8]. The key indicator used to report national progress has been infant mortality. However the Australian Institute of Health and Welfare (AIHW) acknowledges that the precision of these mortality estimates are poor due to the small Australian Indigenous birth cohort and the baseline low mortality risk[9]. Hospital admissions and emergency department presentations reflect morbidities, service provision and care seeking patterns and can be used in combination with mortality data to increase power and precision of analyses[10].

Infants (children aged between 0–11 months) have the highest hospital utilisation rates of all age groups [11], yet there have been no reports of hospital use in Australian Indigenous compared to non-Indigenous infants in the last decade. Causes of infant admission using the International Classification of Disease Version 10 (ICD-10) system are also not widely reported. In 2007 the Northern Territory Department of Health reported that hospital admission rates and the differentials between Indigenous and non-Indigenous infants aged 1–11 months appeared to be increasing[12]. However, there have been no reports of infant hospital admission or emergency department data beyond this period. There are also no reports of admissions within specific socio economic strata. It is not clear if the Australian Federal government ‘Closing the gap’ initiatives have had an effect on reducing the gap between Indigenous and non-Indigenous infant hospital admission and emergency department presentations in the most disadvantaged low income families.

Western Australia (WA) has a large de-identified prospective longitudinal population based data system involving the probabilistic systematic record linkage of total population administrative health datasets[13, 14]. It includes information on maternal and infant characteristics, hospital admission and emergency department presentations including length of stay, cause of hospital admission, Indigenous status and socio economic status.

Our primary objective was to assess the risk of all-cause hospital admission in WA Indigenous and non-Indigenous infants aged under 12 months who were born between 2010 and 2011. Secondary objectives were: (i) to assess the effect of socio economic status on risk of hospital admission; and (ii) to understand the causes of hospital utilisation in Indigenous and non-Indigenous children.

## Methods

### Study setting and data base access

All live births in WA children born from 1 January 2010 to 31 December 2011 were included in this study. Population based linked data from the WA Midwives’ Notification System, Hospital Morbidity Data System, Emergency Department Data Collections, the 2006 Index of Relative Socio-Economic Disadvantage (IRSD)[15] and the Accessibility/ Remoteness Index of Australia (ARIA)[16] were obtained from the Department of Health of Western Australian (DOHWA).

The Midwives’ Notification System includes clinical (infant weight, gestational age, apgar score, multiple birth, gravidity) and socio demographic (baby’s gender, mother’s age, Indigenous status, socioeconomic status, remoteness index) data on all WA live births and stillbirths of more than 20 weeks’ gestation or birth weight greater than 400g which are entered by trained nurses within 48 hours of delivery. The Hospital Morbidity Data System and Emergency Department Data Collections includes data on all hospital admissions to all public and private hospitals and emergency department presentations to all public hospitals in WA. These data are entered by trained medical records staff following the occasion of service. The Australian Bureau of Statistics (ABS) IRSD divides statistical local areas based on the 2006 Australian national census data into quintiles from most disadvantaged (IRSD 1) to least disadvantaged (IRSD 5)[15]. The Accessibility/Remoteness Index of Australia (ARIA)[16] was developed by the Department of Health and Aged Care and is maintained by the AIHW. This index classifies geographic location on the basis of isolation and distance from service centres and health care facilities. ARIA data are split into five categories from least remote (ARIA 1) (major cities) to most remote (ARIA 5) (remote area communities).

The databases were systematically linked by DOHWA data linkage staff using probabilistic matching and de-identified. The final linked database included: date of hospital admission and date of emergency department presentations from the Hospital Morbidity Data System and Emergency Department Data Collections. Maternal ethnicity, maternal age, gravidity, infant age, infant birth weight, gestational age, infant sex, multiple birth, infant health status at birth (Apgar score), IRSD quintile, ARIA level and health region were obtained from the Midwives’ Notification System.

### Definitions

A hospital admission was defined as any (at least one) admission to a WA hospital ward for care including all neonatal nurseries. It excluded the normal hospital stay after birth for well babies. An emergency department presentation was defined as any (at least one) presentation to the emergency department regardless of whether the child was admitted to hospital. The infant period was defined as the period from birth to 11 months of chronological age (i.e. less than 12 months), the neonatal period was from birth to less than one month of age and the post neonatal period was from one month to 11 months of age. An infant was classified as Indigenous if the mother was recorded in the Midwives’ Notification System as Aboriginal and/or Torres Strait Islander[17]. ‘Low socio economic status’ was defined as the two lowest IRSD quintiles (IRSD 1–2). ‘Remote residence’ was defined as the two most remote ARIA categories (ARIA 4–5).

The primary cause of hospitalisation was categorised using the ICD-10 classification system[18] by medical record staff. All hospital admissions were classified using the primary diagnosis at the time of hospital admission but secondary diagnoses or comorbidity data were not available. No data on the diagnosis at the time of the emergency department presentation were available. Preventable causes were defined according to AIHW[19], and adapted for use with infants[20]. Diseases of the respiratory system, digestive system, skin and subcutaneous tissue, ear and mastoid process, infectious and parasitic diseases, nutritional diseases, and injury and poisoning were classified as “preventable”. Perinatal conditions (e.g. prematurity, hypoxic-ischaemic encephalopathy), congenital malformations, chromosomal abnormalities and all other conditions were classified as “non-preventable”. An ‘emergency admission’ was defined as an admission after presentation to the emergency department. An ‘elective admission’ was defined as an admission that was pre-booked and often required a waiting period[21].

### Sample size and data analysis

Our primary outcome measure was the proportion of Indigenous and non-Indigenous infants aged under 12 months who had at least one hospital admission from 2010–2011. We calculated that our study population of almost 63,000 infants would provide 90% power to detect at least a 10% difference in hospital admission risk between Indigenous and non-Indigenous infants. We assumed a 5% significance level, a hospital admission risk of 40% and that the ratio between Indigenous to non-Indigenous infants would be approximately 1:20.

Crude and adjusted logistic regression models were used to examine the effect of Indigenous status and socio economic status on hospital admissions and emergency department presentations in infants aged 0–11 months, neonates (aged 0-<1 month) and post neonates (aged 1–11 months). Effects in IRSD and ARIA strata and specific causes of hospital admission were also assessed. Odds ratios (ORs) and 95% confidence intervals (95% CI) were calculated. Multivariable logistic regression models were constructed *a priori* to adjust for the effect of important explanatory variables: maternal characteristics (maternal age, gravidity), infant factors (gender of child, multiple birth, and birth weight). Data analyses were conducted using STATA 13.1 (StataCorp, USA).

### Ethics

Approvals were obtained from the WA Department of Health Human Research Ethics Committee, The University of Western Australia Human Research Ethics Committee, and the Western Australian Aboriginal Health Ethics Committee (WAAHEC).

## Results

There were 62,965 births in WA from 1 January 2010 to 31 December 2011. Five percent (5.4%, 3,382) of infants were Indigenous and 94.6% (59,583) were non-Indigenous (Table 1).

**Table 1:** Socio demographic characteristics in the study population, 2010–2011.

Characteristics	Total number of childrenn = 62,965	Number of Indigenous children n = 3,382	Number of non- Indigenous children n = 59,583	OR 95% CI	P value
**Socio-economic status (IRSD)**					
Most disadvantaged 1	3,634	1,323 (39.1%)	2,311 (3.9%)	16.33 (15.06,17.72)	<0.001
2	9,670	406 (12.0%)	9,264 (15.6%)	0.75 (0.67,0.83)	<0.001
3	8,126	473 (14.0%)	7,653 (12.8%)	1.11 (1.01,1.23)	0.038
4	17,985	600 (17.7%)	17,385 (29.2%)	0.53 (0.48,0.57)	<0.001
Least disadvantaged 5	22,018	478 (14.1%)	21,540 (36.2%)	0.29 (0.26,0.32)	<0.001
Data missing	1,532	102 (3.0%)	1,430 (2.4%)		
**Geographic location (ARIA)**					
Least remote 1	27,448	574 (17.0%)	26,874 (45.1%)	0.25 (0.23,0.27)	<0.001
2	22,846	664 (19.6%)	22,182 (37.2%)	0.41 (0.28,0.45)	<0.001
3	5,306	486 (14.4%)	4,820 (8.1%)	1.92 (1.74,2.13)	<0.001
4	1,858	180 (5.3%)	1,678 (2.8%)	1.95 (1.67,2.29)	<0.001
Most remote 5	3,975	1,376 (40.7%)	2,599 (4.4%)	15.45 (14.26,16.73)	<0.001
Data missing	1,532	102 (3.0%)	1,430 (2.4%)		
**Maternal Age**					
<20 yrs	2,676	718 (21.2%)	1,958 (3.3%)	7.93 (7.22,8.72)	<0.001
20–24 yrs	9,416	1,121 (33.2%)	8,295 (13.9%)	3.07 (2.84,3.31)	<0.001
25–29 yrs	17,879	817 (24.2%)	17,062 (28.6%)	0.79 (0.73,0.86)	<0.001
30–34 yrs	19,588	459 (13.6%)	19,129 (32.1%)	0.33 (0.30,0.37)	<0.001
35–39 yrs	10,922	221 (6.5%)	10,701 (18.0%)	0.32 (0.28,0.37)	<0.001
40+ yrs	2,477	45 (1.3%)	2,432 (4.1%)	0.32 (0.24,0.43)	<0.001
Data missing	7	1 (0.03%)	6 (0.01%)		
**Gravidity**					
0	19,581	809 (23.9%)	18,772 (31.5%)	0.68 (0.63,0.74)	<0.001
1	19,493	786 (23.2%)	18,707 (31.4%)	0.66 (0.61,0.72)	<0.001
2	11,639	509 (15.1%)	11,130 (18.7%)	0.77 (0.70,0.85)	<0.001
≥3	12,245	1,277 (37.8%)	10,968 (18.4%)	2.69 (2.50,2.89)	<0.001
Data missing	7	1 (0.03%)	6 (0.01%)		
**Child sex**					
Male	32,257	1,789 (52.9%)	30,468 (51.1%)	1.07 (1.00,1.15)	0.046
Female	30,708	1,593 (47.1%)	29,115 (48.9%)	0.93 (0.87,1.00)	0.046
Data missing	0	0 (0.0%)	0 (0.0%)		
**Multiple birth**					
No	61,254	3,311 (97.9%)	57,943 (97.3%)	0.75 (0.59,0.95)	0.020
Yes	1,704	70 (2.1%)	1,634 (2.7%)	1.33 (1.05,1.70)	0.020
Data missing	7	1 (0.03%)	6 (0.01%)		
**Prematurity**					
<32wk	687	83 (2.5%)	604 (1.0%)	2.46 (1.95,3.10)	<0.001
32-36wk	4,586	394 (11.7%)	4,192 (7.0%)	1.75 (1.56,1.95)	<0.001
≥37wk	57,675	2,899 (85.7%)	54,776 (91.9%)	0.53 (0.48,0.59)	<0.001
Data missing	17	6 (0.2%)	11 (0.02%)		
**Birth weight**					
Low birth weight (<2500g)	3,820	440 (13.0%)	3,380 (5.7%)	2.49 (2.24,2.76)	<0.001
Normal birth weight (≥2500-4499g)	58,323	2,909(86.0%)	55,414 (93.0%)	0.46 (0.42,0.51)	<0.001
High birth weight (≥4500g)	821	33 (1.0%)	788 (1.3%)	0.74 (0.52,1.04)	0.085
Data missing	1	0 (0.00%)	1 (0.00%)		
**APGAR 5 score**					
Lowest (least healthy) 1	72	10 (0.3%)	62 (0.1%)	2.85 (1.46,5.56)	<0.001
2	146	12 (0.4%)	134 (0.2%)	1.58 (0.88,2.86)	0.129
3	717	50 (1.5%)	667 (1.1%)	1.33 (0.99,1.77)	0.056
4	3,935	272 (8.0%)	3,663 (6.1%)	1.34 (1.18,1.52)	<0.001
Highest (most healthy) 5	58,042	3,032 (89.7%)	55,010 (92.3%)	0.73 (0.65,0.81)	<0.001
Data missing	53	6 (0.2%)	47 (0.1%)		

IRSD = Index of Relative Socio-Economic Disadvantage ARIA = Accessibility/ Remoteness Index of Australia, OR = odds ratio, 95% CI = 95% confidence interval

Thirty nine percent (39.1%, 1323) of Indigenous and 3.9% (2,311) of non-Indigenous infants were in the most disadvantaged quintile (IRSD 1) (Table 1). Forty percent (40.7%, 1376) of Indigenous and 4.4% (2,599) of non-Indigenous infants lived in the most remote area (ARIA 1) (Table 1) (S1 Appendix).

There were 28,960 hospital admissions in 18,879 infants in the first 12 months of life. Indigenous infants were 1.7 times more likely to be admitted to hospital at least once (44.0%) compared to non-Indigenous infants (29.2%) (aOR 1.71, 95% CI 1.58–1.85) (Table 2). Ten percent (10.4%, 352) of Indigenous infants had three or more admissions to hospital in their first year of life compared to three percent (3.1%, 1834) of non-Indigenous infants (aOR 2.26, 95% CI 1.96–2.61) (Table 2). Risk of hospital admission was slightly higher in the neonatal period (0-<1 month) (19.02%, 11,977) than the post neonatal period (1-11months) (15.9%, 9,993). The effect of Indigenous status on hospital admission greater in the post neonatal period (aOR 1.87, 95% CI 1.72–2.03) than the neonatal period (aOR 1.25, 95% CI 1.14–1.38) (S1 Appendix).

**Table 2:** Effect of Indigenous status on hospital utilisation, 2010–2011.

	Number of Indigenous infants with at least one hospital admission or emergency department presentation n = 3,382	Number of non-Indigenous infants with at least one hospital admission or emergency department presentation n = 59,583	OR (95% CI)	p value	aOR* (95% CI)	p value
All cause hospitalisations						
at least 1	1,487 (44.0%)	17,392 (29.2%)	1.90 (1.77,2.04)	<0.001	1.71 (1.58,1.85)	<0.001
at least 2	708 (20.9%)	5,079 (8.5%)	2.84 (2.60,3.10)	<0.001	1.95 (1.76,2.17)	<0.001
at least 3	352 (10.4%)	1,834 (3.1%)	3.66 (3.25,4.12)	<0.001	2.26 (1.96,2.61)	<0.001
All cause emergency department presentations						
at least 1	2,388 (70.6%)	25,238 (42.4%)	3.27 (3.03,3.53)	<0.001	2.15 (1.98,2.33)	<0.001
at least 2	1,774 (52.5%)	12,006 (20.2%)	4.37 (4.07,4.69)	<0.001	2.61 (2.42,2.82)	<0.001
at least 3	1310 (38.7%)	6,002 (10.1%)	5.62 (5.22,6.06)	<0.001	3.04 (2.80,3.31)	<0.001

OR = odds ratio, aOR = adjusted odds ratio, 95% CI = 95% confidence interval

* Adjusted for IRSD (Index of Relative Socio-Economic Disadvantage), maternal age, gravidity, sex of child, multiple birth, birth weight

The risk of hospital admission was significantly higher for infants of teenage mothers (aOR 1.28, 95% CI 1.17–1.41, p value <0.001) and mothers ≥40 years of age (aOR 1.19, 95% CI 1.08–1.31, p-value <0.001) (S1 Appendix).

Socio economic status (IRSD) had little influence on the risk of hospitalisation (Table 3). The risk of hospital admission in the most disadvantaged (IRSD 1) infants (35.7%) was similar to the risk in the least disadvantaged (IRSD 5) infants (30.6%) (aOR 1.04, 95% CI 0.96–1.13, p value 0.356). There was weak evidence of a dose response of increasing risk of hospital admission with increasing levels of disadvantage in Indigenous (p = 0.017 for trend) and non-Indigenous (p = 0.020 for trend) (Table 3). The risk of hospital admission in the least disadvantaged Indigenous infants (40.2%) was greater than the risk in the most disadvantaged non-Indigenous infants (28.9%) (Table 3). In the three least disadvantaged quintiles (IRSD 1–3), Indigenous infants were still 1.5 times more likely to be admitted to hospital than non-Indigenous infants (aOR 1.54, 95% CI 1.38–1.72). Effects of IRSD were greater in the post neonatal (p < 0.001 for trend) than the neonatal (p = 0.054 for trend) periods (S1 Appendix).

**Table 3:** Effect of socio economic quintile and geographic location on hospital utilisation in Indigenous and non Indigenous infants, 2010–2011.

	Indigenous			Non Indigenous		
	Total no of Indigenous infants n = 3,382	Number of indigenous infants with at least one hospital admission n = 1,487	aOR* (95% CI)	Total no of non-Indigenous infants n = 59,583	Number of non-Indigenous infants with at least one hospital admission n = 17,392	aOR* (95% CI)
Socio economic status (IRSD)						
Most disadvantaged 1	1,323	629 (47.5%)	1.30 (1.04,1.63)	2,311	668 (28.9%)	0.98 (0.89,1.09)
2	406	168 (41.4%)	1.02 (0.77,1.35)	9,264	2,690 (29.0%)	0.93 (0.88,0.98)
3	473	187 (39.5%)	1.00 (0.76,1.31)	7,653	2,045 (26.7%)	0.85 (0.80,0.90)
4	600	263 (43.8%)	1.13 (0.88,1.46)	17,385	5,005 (28.8%)	0.94 (0.90,0.99)
Least disadvantaged 5	478	192 (40.2%)	1.00	21,540	6,537 (30.4%)	1.00
			P value trend 0.017			P value trend 0.020
Geographic location (ARIA)					
Most remote 5	1,376	634 (46.1%)	1.34 (1.09,1.64)	2,599	652 (25.1%)	0.89 (0.81,0.98)
4	180	61 (33.9%)	0.83 (0.57,1.19)	1,678	457 (27.2%)	0.97 (0.87,1.09)
3	486	210 (43.2%)	1.14 (0.89,1.48)	4,820	1,387 (28.8%)	1.07 (1.00,1.15)
2	664	302 (45.5%)	1.32 (1.04,1.67)	22,182	6,619 (29.8%)	1.09 (1.04,1.13)
Least remote 1	574	232 (40.4%)	1.00	26,874	7,830 (29.1%)	1.00
			P value trend 0.051			P value trend 0.591
		Number of Indigenous infants with at least one emergency department presentation n = 2,388			Number of non-Indigenous infants with at least one emergency department presentation n = 25,238	
Socio economic status (IRSD)					
Most disadvantaged 1	1,323	983 (74.3%)	1.36 (1.08,1.71)	2,311	1,426 (61.7%)	2.43 (2.22,2.66)
2	406	271 (66.8%)	0.95 (0.72,1.26)	9,264	4,145 (44.7%)	1.23 (1.17,1.30)
3	473	336 (71.0%)	1.17 (0.89,1.55)	7,653	3,608 (47.1%)	1.35 (1.28,1.43)
4	600	402 (67.0%)	0.93 (0.72,1.21)	17,385	7,321 (42.1%)	1.11 (1.06,1.16)
Least disadvantaged 5	478	323 (67.6%)	1.00	21,540	8,132 (37.8%)	1.00
			P value trend 0.001			P value trend <0.001
Geographic location (ARIA)					
Most remote 5	1,376	1,035 (75.2%)	1.68 (1.36,2.08)	2,599	1,592 (61.3%)	2.52 (2.31,2.73)
4	180	139 (77.2%)	1.95 (1.32,2.88)	1,678	942 (56.1%)	1.92 (1.73,2.12)
3	486	353 (72.6%)	1.46 (1.12,1.90)	4,820	2,583 (53.6%)	1.77 (1.66,1.88)
2	664	416 (62.7%)	0.92 (0.73,1.16)	22,182	9,365 (42.2%)	1.10 (1.06,1.15)
Least remote 1	574	372 (64.8%)	1.00	26,874	10,150 (37.8%)	1.00
			P value trend <0.001			P value trend <0.001

IRSD = Index of Relative Socio-Economic Disadvantage ARIA = Accessibility/ Remoteness Index of Australia, OR = odds ratio, aOR = adjusted odds ratio, 95% CI = 95% confidence interval

* Adjusted for maternal age, gravidity, sex of child, multiple birth, birth weight

Geographic location had little effect on the risk of hospital admission in Indigenous and non-Indigenous infants (Table 3). The risk of hospital admission in the most remote (ARIA 5) infants (32.4%) was similar to the risk in the least remote (ARIA 1) infants (29.4%) (aOR 0.98, 95% CI 0.91–1.07, p value 0.694). There was no evidence of increasing risk of hospital admission with increasing levels of remoteness in Indigenous (p = 0.051 for trend) and non-Indigenous (p = 0.591 for trend) (Table 3). The risk of hospital admission in the least remote Indigenous children (40.4%) was greater than the most remote non-Indigenous children (25.1%) (Table 3). In the three least remote areas (ARIA 1–3) Indigenous infants were still 1.6 times more likely to be admitted to hospital than non-Indigenous infants (aOR 1.60, 95% CI 1.44–1.77).

Indigenous infants were twofold more likely to present to the emergency department at least once (70.6%) compared to non-Indigenous infants (42.4%) (aOR 2.15, 95% CI 1.98–2.33) (Table 2). Effects of socio economic status and geographic location on emergency department presentations were similar to the effects on hospital admission (Tables 2 and 3) (S1 Appendix).

Fifty nine percent (59.0%) of admissions in Indigenous infants and 31.2% of admissions in non-Indigenous infants were classified as ‘preventable’ (diseases of the respiratory system; infectious and parasitic diseases; digestive system; skin and subcutaneous tissue; ear and mastoid process; nutritional diseases; injury and poisoning) (Table 4). Risk of preventable hospital admission was twofold higher in Indigenous compared to non Indigenous infants (aOR 2.12, 95% CI 1.88–2.39) (Table 4). Risk of perinatal disorders was lower in Indigenous (657, 44.2%) compared to non Indigenous (9,972, 57.3%) infants (aOR 0.68, 95% CI 0.60–0.77).

**Table 4:** ICD 10 classification of primary cause of hospital admissions in the study population by Indigenous status, 2010–2011.

Primary cause of hospital admission	Total number of infants	Number of Indigenous infants	Number of non-Indigenous infants	OR (95% CI; p value)	aOR** (95% CI; p value)
	18,879	1,487	17,392		
**Preventable causes**					
Respiratory system	3,146 (16.7%)	580 (39.0%)	2566 (14.8%)	3.69 (3.30,4.13; <0.001)	2.20 (1.94,2.50; <0.001)
Infectious and parasitic diseases	1,550 (8.2%)	240 (16.1%)	1310 (7.5%)	2.36 (2.04,2.74; <0.001)	1.78 (1.50,2.11; <0.001)
Digestive system	797 (4.2%)	50 (3.4%)	747 (4.3%)	0.78 (0.58,1.04; 0.087)	0.59 (0.43,0.81; 0.001)
Skin and subcutaneous tissue	343 (1.8%)	80 (5.4%)	263 (1.5%)	3.70 (2.87,4.78; <0.01)	3.48 (2.56,4.72; <0.001
Ear and mastoid process	344 (1.8%)	58 (3.9%)	286 (1.6%)	2.43 (1.8,3.24; <0.001)	2.65 (1.90,3.69; <0.001)
Nutritional diseases	105 (0.6%)	11 (0.7%)	94 (0.5%)	1.37 90.73,2.57; 0.323)	1.18 (0.59,2.35; 0.641)
Injury and poisoning	684 (3.6%)	68 (4.6%)	616 (3.5%)	1.31 (1.01,1.69; 0.042)	1.20 (0.91,1.59; 0.206)
**Total preventable causes**	**6,300 (33.4%)**	**878 (59.0%)**	**5422 (31.2%)**	**3.18 (2.86,3.55; <0.001)**	**2.12 (1.88,2.39; <0.001)**
**Non preventable causes**					
Perinatal conditions	10,629 (56.3%)	657 (44.2%)	9972 (57.3%)	0.59 (0.53,0.66; <0.001)	0.68 (0.60,0.77; <0.001)
Congenital malformations, deformations and chromosomal abnormalities	1,336 (7.1%)	51 (3.4%)	1285 (7.4%)	0.45 (0.33,0.59; <0.001)	0.49 (0.36,0.66; <0.001)
Other	4,516 (23.9%)	352 (23.7%)	4164 (23.9%)	0.99 (0.87,1.12; 0.815)	0.94 (0.82,1.08; 0.401)
**Total non preventable causes**	**14,855 (78.7%)**	**940 (63.2%)**	**13915 (80.0%)**	**0.43 (0.38,0.48; <0.001)**	**0.57 (0.50,0.65; <0.001)**

*Children have been counted only once per condition. Children may be included in more than one condition if they had multiple admissions in their first year of life

OR = odds ratio, aOR = adjusted odds ratio, 95% CI = 95% confidence interval

** Adjusted for IRSD (Index of Relative Socio-Economic Disadvantage), maternal age, gravidity, sex of child, multiple birth, birth weight

Seventy percent (70.6%, 2388) of Indigenous infants were classified as having an emergency admission compared to 42.4% (25,238) of non-Indigenous infants (aOR 2.15, 95% CI 1.98–2.33) (S1 Appendix).

## Discussion

We report important differences in hospital utilisation between Indigenous and non-Indigenous children in WA. In our study 70% of Indigenous infants presented to hospital emergency departments and 40% were admitted in their first year of life. Risks of hospital admission and emergency department presentation were 1.5 to three fold greater in Indigenous compared to non-Indigenous infants and were highest in the youngest, most disadvantaged, remote area infants.

The AIHW and all Australian states and territories hold age specific hospital admission data from birth to adulthood[21]. Infants aged 0–11 months have the highest risk of admission of all age groups, yet surprisingly there have been no publications in the past decade which have examined all cause hospital utilisation in Australian Indigenous and non-Indigenous infants in their first year of life. In 2002 the Northern Territory Department of Health reported all-cause post neonatal hospital admission rates (72%) that were much higher than the rates reported in our study (40%).[12] From 2008–2013 studies from eastern Australia (Victoria[22] and New South Wales[20]) and WA[23] reported high risks of all cause emergency department presentation in Indigenous children aged 0–4 years. There are limited data on ‘all-cause’ hospital utilisation in children under five years from Indigenous populations in Australia or other countries. In the Northern Territory from 1992 to 2008 there was no improvement in the differential in ‘all-cause’ hospitalisation separation rates between Indigenous and non Indigenous children aged under 5 years. The risk was approximately two fold greater in Indigenous compared to non Indigenous children[24].

Our study provides evidence that the effect of Indigenous status on hospital utilisation is stronger than the effect of socio economic status or geographic location in WA infants. The risk of hospital admission in the least disadvantaged urban Indigenous infants was greater than the risk in the most disadvantaged remote area non-Indigenous infants. The only study that has examined the effect of geographic location on hospital utilisation in Indigenous and non-Indigenous children was conducted in eastern Australia (Victoria)[22]. This study reported that rural Indigenous children had a threefold greater risk of emergency department presentation than rural non-Indigenous children but urban city Indigenous children had a very similar risk to urban non-Indigenous children[22]. The high risks in our urban infants may be due to greater mobility of Indigenous families between WA urban and rural areas. To our knowledge no studies have been published that have examined the effect of socio economic status on hospital utilisation in Indigenous infants in Australia or other countries.

Our study also appears to be the first that has reported on the burden of preventable hospital admissions in Indigenous infants in Australia. The burden includes respiratory disease, gastroenteritis, ear disease, skin infections, other infections and injury. Our data indicate that over 50% of hospital admissions in Indigenous infants are preventable and twice as high as in non-Indigenous infants. Similarly high rates are reported in Australian Indigenous adults[25], but no other studies appear to have been published that have examined the burden of preventable hospital admissions in Indigenous infants under 12 months of age.

Our study had some limitations. Hospital utilisation data provide important information on serious acute and chronic illnesses but rates can change with care seeking practices, resourcing and admission policy. Our study was observational and was only able to report associations not causality. We adjusted for all available potential confounding factors but did not have a measure of maternal illness or education. Our socio economic data was based primarily on the AIHW IRSD quintiles which can cause misclassification when applied at an individual level[15]. This may be the reason for the weak associations that we reported between hospital utilisation and socio economic status. We had data on the number of teenage mothers and adjusted for this in our multivariable analyses. However, we did not have access to data on underlying social conditions that may be associated with the preventable causes of hospital utilisation, for example housing and infrastructure issues[26].

We relied on hospital coding of Indigenous status and it is well known that missing or incorrect Indigenous status may lead to under-estimation of risk[27, 28]. However, we reported highly significant effects of Indigenous status on hospital utilisation and any potential misclassification is likely to have biased towards the null. Our cause specific hospitalisation data were limited to primary cause of hospitalisation. These data are considered to be highly accurate,[13, 14, 29] because the Hospital Morbidity Data System uses the World Health Organisation ICD 10 coding system[18] and highly trained coders. The Midwives’ Notification System also uses clear definitions that are based on Australian standard definitions[30]. It is reported to have a very high level of completion and clinical certainty[31, 32]. Our emergency department presentations were also recorded in a clearly defined patient administration system (‘EDIS’)[33, 34]. This system is considered by Emergency Department staff to be highly reliable though formal documentation of its accuracy is not available. In contrast, the accuracy of cause specific emergency department data has been questioned[23]. This was the reason we did not include cause specific emergency department data in this study.

Other strengths of our study included the population based prospective data collection and large sample size of over 60,000 infants. Reverse causality was unlikely and there were little missing data. Data were ascertained by trained midwifes and medical records staff and no self-report was included.

Our study has implications for program and policy development. WA Indigenous children had high hospital utilisation regardless of their socio economic status and geographic location indicating that WA health services should prioritise Indigenous children no matter where they live. More than half of the hospital presentations in Indigenous infants were preventable. The high emergency department presentations in remote area Indigenous infants are of particular concern. Reasons include high levels of morbidity and lack of ‘out of hours’ primary care services. It is likely that a number of the emergency department presentations were due to problems that could be managed in general practice services, other primary care centres and other treatment facilities[35–37]. The WA primary care system appears to be failing many Indigenous children in both urban and remote WA[38–40]. Improved access to primary care centres (including evening and weekend opening times, transport for families, electronic recall and reminder systems) and quality of care (including cultural security and protocols for assessment and referral of young infants) is needed. There are also few primary care data sets that can be used to report health service use in Indigenous children. These are needed so service improvements can be measured and monitored.

## Supporting Information

S1 Appendix Socio demographic characteristics and hospital utilisation in the study population, 2010–2011 (**Table A**). Effect of socio demographic characteristics on hospital utilisation in the study population, 2010–2011 (**Table B**). Risk of hospital admission in Indigenous and non-Indigenous infants aged 0-<1m (neonates) by socio economic status, 2010–2011 (**Table C**). Risk of emergency department presentation in Indigenous and non-Indigenous infants aged 0-<1m (neonates) by socio economic status, 2010–2011 (**Table D**). Risk of hospital admission in Indigenous and non-Indigenous infants aged 1-11m (post neonates) by socio economic status, 2010–2011 (**Table E**). Risk of emergency department presentation in Indigenous and non-Indigenous infants aged 1-11m (post neonates) by socio economic status., 2010–2011 (**Table F**).
(DOCX)
