# Supplementary material for: Hospital Utilisation in Indigenous and Non-Indigenous Infants under 12 Months of Age in Western Australia, Prospective Population Based Data Linkage Study
Source: PLoS One. 2016 Apr 27;11(4):e0154171. doi: 10.1371/journal.pone.0154171 (PMC4847930; doi:10.1371/journal.pone.0154171)
Supplement: S1 Appendix — Socio demographic characteristics and hospital utilisation in the study population, 2010–2011 (Table A). Effect of socio demographic characteristics on hospital utilisation in the study population, 2010–2011 (Table B). Risk of hospital admission in Indigenous and non-Indigenous infants aged 0-<1m (neonates) by socio economic status, 2010–2011 (Table C). Risk of emergency department presentation in Indigenous and non-Indigenous infants aged 0-<1m (neonates) by socio economic status, 2010–2011 (Table D). Risk of hospital admission in Indigenous and non-Indigenous infants aged 1-11m (post neonates) by socio economic status, 2010–2011 (Table E). Risk of emergency department presentation in Indigenous and non-Indigenous infants aged 1-11m (post neonates) by socio economic status., 2010–2011 (Table F). (DOCX) [file pone.0154171.s001.docx]

**Table A Socio demographic characteristics and hospital utilisation in the study population, 2010-2011**

|  |  |  |  | **All cause hospital admissions <12months of age** | | | **All cause emergency department presentations <12months of age** | | |
| --- | --- | --- | --- | --- | --- | --- | --- | --- | --- |
| **Characteristics** | Total number of children  62,965 | Number of Indigenous children  3,382 | Number of Non- Indigenous children  59,58 | Total number of children with at least one admission  18,879 | Number of Indigenous children with at least one admission  1,487 | Number of non-Indigenous children with at least one admission  17,392 | Total number of children with at least one presentation  27,626 | Number of Indigenous children with at least one presentation  2,388 | Number of non-Indigenous children with at least one presentation  25,238 |
| **Socio-economic status (IRSD quintile)** |  |  |  |  |  |  |  |  |  |
| Most disadvantaged1 | 3,634 | 1,323 (39.1%) | 2,311 (3.9%) | 1,297 (35.7%) | 629 (47.5%) | 668 (28.9%) | 2,409 (66.3%) | 983 (74.3%) | 1,426 (61.7%) |
| 2 | 9,670 | 406 (12.0%) | 9,264 (15.6%) | 2,858 (29.6%) | 168 (41.4%) | 2,690 (29.0%) | 4,416 (45.7%) | 271 (66.8%) | 4,145 (44.7%) |
| 3 | 8,126 | 473 (14.0%) | 7,653 (12.8%) | 2,232 (27.5%) | 187 (39.5%) | 2,045 (26.7%) | 3,944 (48.5%) | 336 (71.0%) | 3,608 (47.1%) |
| 4 | 17,985 | 600 (17.7%) | 17,385 (29.2%) | 5,268 (29.3%) | 263 (43.8%) | 5,005 (28.8%) | 7,723 (42.9%) | 402 (67.0%) | 7,321 (42.1%) |
| Least disadvantaged 5 | 22,018 | 478 (14.1%) | 21,540 (36.2%) | 6,729 (30.6%) | 192 (40.2%) | 6,537 (30.4%) | 8,455 (38.4%) | 323 (67.6%) | 8,132 (37.8%) |
| Data missing | 1,532 | 102 (3.0%) | 1,430 (2.4%) | 495 (2.6%) | 48 (3.2%) | 447 (2.6%) | 679 (2.5%) | 73 (3.1%) | 606 (2.4%) |
| **Remoteness (ARIA)** |  |  |  |  |  |  |  |  |  |
| Least remote 1 | 27,448 | 574 (17.0%) | 26,874 (45.1%) | 8,062 (29.4%) | 232 (40.4%) | 7,830 (29.1%) | 10,522 (38.3%) | 372 (64.8%) | 10,150 (37.8%) |
| 2 | 22,846 | 664 (19.6%) | 22,182 (37.2%) | 6,921 (30.3%) | 302 (45.5%) | 6,619 (29.8%) | 9,781 (42.8%) | 416 (62.7%) | 9,365 (42.2%) |
| 3 | 5,306 | 486 (14.4%) | 4,820 (8.1%) | 1,597 (30.1%) | 210 (43.2%) | 1,387 (28.8%) | 2,936 (55.3%) | 353 (72.6%) | 2,583 (53.6%) |
| 4 | 1,858 | 180 (5.3%) | 1,678 (2.8%) | 518 (27.9%) | 61 (33.9%) | 457 (27.2%) | 1,081 (58.2%) | 139 (77.2%) | 942 (56.1%) |
| Most remote 5 | 3,975 | 1,376 (40.7%) | 2,599 (4.4%) | 1,286 (32.4%) | 634 (46.1%) | 652 (25.1%) | 2,627 (66.1%) | 1,035 (75.2%) | 1,592 (61.3%) |
| Data missing | 1,532 | 102 (3.0%) | 1,430 (2.4%) | 495 (2.6%) | 48 (3.2%) | 447 (2.6%) | 679 (2.5%) | 73 (3.1%) | 606 (2.4%) |
| **Maternal Age** |  |  |  |  |  |  |  |  |  |
| <20 yrs | 2,676 | 718 (21.2%) | 1,958 (3.3%) | 1,009 (37.7%) | 334 (46.5%) | 675 (34.5%) | 1,765 (66.0%) | 548 (76.3%) | 1,217 (62.2%) |
| 20-24 yrs | 9,416 | 1,121 (33.2%) | 8,295 (13.9%) | 2,926 (31.1%) | 488 (43.5%) | 2,438 (29.4%) | 5,262 (55.9%) | 815 (72.7%) | 4,447 (53.6%) |
| 25-29 yrs | 17,879 | 817 (24.2%) | 17,062 (28.6%) | 5,089 (28.5%) | 339 (41.5%) | 4,750 (27.8%) | 8,004 (44.8%) | 544 (66.6%) | 7,460 (43.7%) |
| 30-34 yrs | 19,588 | 459 (13.6%) | 19,129 (32.0%) | 5,686 (29.0%) | 196 (42.7%) | 5,490 (28.7%) | 7,604 (38.8%) | 304 (66.2%) | 7,300 (38.2%) |
| 35-39 yrs | 10,922 | 221 (6.5%) | 10,701 (18.0%) | 3,327 (30.5%) | 110 (49.8%) | 3,217 (30.1%) | 4,069 (37.3%) | 149 (67.4%) | 3,920 (36.6%) |
| 40+ yrs | 2,477 | 45 (1.3%) | 2,432 (4.1%) | 835 (33.7%) | 19 (42.2%) | 816 (33.6%) | 919 (37.1%) | 28 (62.2%) | 891 (36.6%) |
| Data missing | 7 | 1 (0.03%) | 6 (0.01%) | 7 (0.04%) | 1 (0.1%) | 6 (0.03%) | 3 (0.01%) | 0 (0.0%) | 3 (0.01%) |
| **Gravidity** |  |  |  |  |  |  |  |  |  |
| 0 | 19,581 | 809 (23.9%) | 18,772 (31.5%) | 6,150 (31.4%) | 404 (50.0%) | 5,746 (30.6%) | 8,687 (44.4%) | 639 (79.0%) | 8,048 (42.9%) |
| 1 | 19,493 | 786 (23.2%) | 18,707 (31.4%) | 5,488 (28.2%) | 319 (40.6%) | 5,169 (27.6%) | 8,278 (42.5%) | 544 (69.2%) | 7,734 (41.3%) |
| 2 | 11,639 | 509 (15.1%) | 11,130 (18.7%) | 3,367 (28.9%) | 224 (44.0%) | 3,143 (28.2%) | 4,977 (42.8%) | 361 (70.9%) | 4,616 (41.5%) |
| ≥3 | 12,245 | 1,277 (37.8%) | 10,968 (18.4%) | 3,867 (31.6%) | 539 (42.2%) | 3,328 (30.3%) | 5,681 (46.4%) | 844 (66.1%) | 4,837 (44.1%) |
| Data missing | 7 | 1 (0.03%) | 6 (0.01%) | 7 (0.04%) | 1 (0.1%) | 6 (0.03%) | 3 (0.01%) | 0 (0.0%) | 3 (0.01%) |
| **Child sex** |  |  |  |  |  |  |  |  |  |
| Male | 32,257 | 1,789 (52.9%) | 30,468 (51.1%) | 10,812 (33.52%) | 843 (47.1%) | 9,969 (32.7%) | 14,874 (46.1%) | 1,304 (72.9%) | 13,570 (44.5%) |
| Female | 30,708 | 1,593 (47.1%) | 29,115 (48.9%) | 8,067 (26.27%) | 644 (40.4%) | 7,423 (25.5%) | 12,752 (41.5%) | 1,084 (68.1%) | 11,668 (40.1%) |
| Data missing | 0 | 0 (0.0%) | 0 (0.0%) | 0 (0.0%) | 0 (0.0%) | 0 (0.0%) | 0 (0.0%) | 0 (0.0%) | 0 (0.0%) |
| **Multiple birth** |  |  |  |  |  |  |  |  |  |
| No | 61,254 | 3,311 (97.9%) | 57,943 (97.3%) | 17,586 (28.7%) | 1,430 (43.2%) | 16,156 (27.9%) | 26,942 (44.0%) | 2,335 (70.5%) | 24,607 (42.5%) |
| Yes | 1,704 | 70 (2.1%) | 1,634 (2.7%) | 1,286 (75.5%) | 56 (80.0%) | 1,230 (75.3%) | 681 (40.0%) | 53 (75.7%) | 628 (38.4%) |
| Data missing | 7 | 1 (0.03%) | 6 (0.01%) | 7 (0.04%) | 1 (0.1%) | 6 (0.03%) | 3 (0.01%) | 0 (0.00%) | 3 (0.01%) |
| **Prematurity** |  |  |  |  |  |  |  |  |  |
| <32wk | 687 | 83 (2.5%) | 604 (1.0%) | 657 (95.6%) | 79 (95.2%) | 578 (95.7%) | 373 (54.3%) | 62 (74.7%) | 311 (51.5%) |
| 32-36wk | 4,586 | 394 (11.7%) | 4,192 (7.0%) | 3,478 (75.9%) | 295 (74.9%) | 3,183 (75.9%) | 2,291 (50.0%) | 293 (74.4%) | 1,998 (47.7%) |
| >=37wk | 57,675 | 2,899 (85.7%) | 54,776 (91.9%) | 14,729 (25.5%) | 1,108 (38.2%) | 13,621 (24.9%) | 24,952 (43.3%) | 2,029 (70.0%) | 22,923 (41.9%) |
| Data missing | 17 | 6 (0.2%) | 11 (0.02%) | 15 (0.1%) | 5 (0.3%) | 10 (0.1%) | 10 (0.04%) | 4 (0.2%) | 6 (0.02%) |
| **Birth weight** |  |  |  |  |  |  |  |  |  |
| Low birth weight (<2500g) | 3,820 | 440 (13.0%) | 3,380 (5.7%) | 3,138 (82.2%) | 368 (83.6%) | 2,770 (82.0%) | 1,903 (49.8%) | 337 (76.6%) | 1,566 (46.3)% |
| Normal birth weight (≥2500g) | 59,144 | 2,942 (87.0%) | 56,202 (94.3%) | 15,740 (26.6%) | 1,119 (38.0%) | 14,621 (26.0%) | 25,722 (43.5%) | 2,051 (69.7%) | 23,671 (42.2)% |
| Data missing | 1 | 0 (0.0%) | 1 (0.002%) | 1 (0.01%) | 0 (0.0%) | 1 (0.01%) | 1 (0.002%) | 0 (0.0%) | 1 (0.002)% |
| **APGAR 5 score** |  |  |  |  |  |  |  |  |  |
| Lowest (least healthy) 1 | 72 | 10 (0.3%) | 62 (0.1%) | 38 (52.8%) | 3 (30.0%) | 35 (56.5%) | 19 (26.4%) | 5 (50.0%) | 14 (22.6)% |
| 2 | 146 | 12 (0.35%) | 134 (0.2%) | 124 (84.9%) | 11 (91.7%) | 113 (84.3%) | 76 (52.1%) | 10 (83.3%) | 66 (49.3)% |
| 3 | 717 | 50 (1.48%) | 667 (1.1%) | 547 (76.3%) | 41 (82.0%) | 506 (75.9%) | 378 (52.7%) | 36 (72.0%) | 342 (51.3)% |
| 4 | 3,935 | 272 (8.04%) | 3,663 (6.2%) | 2,384 (60.6%) | 195 (71.7%) | 2,189 (59.8%) | 1,964 (49.9%) | 204 (75.0%) | 1,760 (48.1)% |
| Highest (most healthy) 5 | 58,042 | 3,032 (89.7%) | 55,010 (92.3%) | 15,767 (27.2%) | 1,233 (40.7%) | 14,534 (26.4%) | 25,168 (43.4%) | 2,130 (70.3%) | 23,038 (41.9)% |
| Data missing | 53 | 6 (0.2%) | 47 (0.1%) | 19 (0.1%) | 4 (0.3%) | 15 (0.1%) | 21 (0.1%) | 3 (0.1%) | 18 (0.1%) |

IRSD = Index of Relative Socio-Economic Disadvantage, ARIA = Accessibility/ Remoteness Index of Australia

**Table B Effect of socio demographic characteristics on hospital utilisation in the study population, 2010-2011**

| **Characteristics** | **All cause hospitalisations <12months of age** | | | |  | **All cause emergency department presentations <12months of age** | | | | |
| --- | --- | --- | --- | --- | --- | --- | --- | --- | --- | --- |
|  | **Number of children** | unadjusted  OR (95% CI) | *p value* | multivariable  OR (95% CI)* | *p value* | **Number of children** | unadjusted  OR (95% CI) | *p value* | multivariable  OR (95% CI)* | *p value* |
| **Indigenous status** |  |  |  |  |  |  |  |  |  |  |
| Indigenous | 1,487 (44.0%) | 1.90 (1.77,2.04) | <0.001 | 1.71 (1.58,1.85) | <0.001 | 2,388 (70.6%) | 3.27 (3.03,3.53) | <0.001 | 2.15 (1.98,2.33) | <0.001 |
| Non-Indigenous | 17,392 (29.2%) | 1.00 |  | 1.00 |  | 25,238 (42.4%) | 1.00 |  | 1.00 |  |
|  |  |  |  |  |  |  |  |  |  |  |
| **Socio-economic status (IRSD quintile)** |  |  |  |  |  |  |  |  |  |  |
| Most disadvantaged 1 | 1,297 (35.7%) | 1.26 (1.17,1.36) | <0.001 | 1.04 (0.96,1.13) | 0.356 | 2,409 (66.3%) | 3.15 (2.93,3.40) | <0.001 | 2.20 (2.03,2.38) | <0.001 |
| 2 | 2,858 (29.6%) | 0.95 (0.90,1.00) | 0.073 | 0.93 (0.88,0.98) | 0.012 | 4,416 (45.7%) | 1.35 (1.28,1.42) | <0.001 | 1.23 (1.17,1.29) | <0.001 |
| 3 | 2,232 (27.5%) | 0.86 (0.81,0.910 | <0.001 | 0.86 (0.81,0.91) | <0.001 | 3,944 (48.5%) | 1.51 (1.44,1.59) | <0.001 | 1.36 (1.29,1.43) | <0.001 |
| 4 | 5,268 (29.3%) | 0.94 (0.90,0.98) | 0.006 | 0.95 (0.91,0.99) | 0.030 | 7,723 (42.9%) | 1.21 (1.16,1.26) | <0.001 | 1.11 (1.07,1.16) | <0.001 |
| Least disadvantaged 5 | 6,729 (30.6%) | 1.00 |  | 1.00 |  | 8,455 (38.4%) | 1.00 |  | 1.00 |  |
|  |  |  |  |  |  |  |  |  |  |  |
| **Remoteness (ARIA)** |  |  |  |  |  |  |  |  |  |  |
| Least remote 1 | 8,062 (29.4%) | 1.00 |  | 1.00 |  | 10,522 (38.3%) | 1.00 |  | 1.00 |  |
| 2 | 6,921 (30.3%) | 1.04 (1.01,1.09) | 0.024 | 1.09 (1.05,1.14) | <0.001 | 9,781 (42.8%) | 1.20 (1.16,1.25) | <0.001 | 1.10 (1.06,1.14) | <0.001 |
| 3 | 1,597 (30.1%) | 1.04 (0.97,1.10) | 0.288 | 1.07 (1.00,1.14) | 0.055 | 2,936 (55.3%) | 1.99 (1.88,2.11) | <0.001 | 1.76 (1.66,1.88) | <0.001 |
| 4 | 518 (27.9%) | 0.93 (0.84,1.03) | 0.171 | 0.94 (0.84,1.05) | 0.264 | 1,081 (58.2%) | 2.24 (2.03,2.46) | <0.001 | 1.94 (1.76,2.14) | <0.001 |
| Most remote 5 | 1,286 (32.4%) | 1.15 (1.07,1.24) | <0.001 | 0.98 (0.91,1.07) | 0.694 | 2,627 (66.1%) | 3.13 (2.92,3.36) | <0.001 | 2.37 (2.20,2.55) | <0.001 |
|  |  |  |  |  |  |  |  |  |  |  |
| **Maternal Age** |  |  |  |  |  |  |  |  |  |  |
| <20 yrs | 1,009 (37.7%) | 1.52 (1.40,1.66) | <0.001 | 1.28 (1.17,1.41) | <0.001 | 1,765 (66.0%) | 2.39 (2.20,2.60) | <0.001 | 2.05 (1.88,2.24) | <0.001 |
| 20-24 yrs | 2,926 (31.1%) | 1.13 (1.07,1.20) | <0.001 | 1.08 (1.01,1.14) | 0.012 | 5,262 (55.9%) | 1.56 (1.48,1.64) | <0.001 | 1.47 (1.40,1.55) | <0.001 |
| 25-29 yrs | 5,089 (28.5%) | 1.00 |  | 1.00 |  | 8,004 (44.8%) | 1.00 |  | 1.00 |  |
| 30-34 yrs | 5,686 (29.0%) | 1.03 (0.98,1.08) | 0.228 | 1.03 (0.98,1.08) | 0.244 | 7,604 (38.8%) | 0.78 (0.75,0.82) | <0.001 | 0.80 (0.77,0.83) | <0.001 |
| 35-39 yrs | 3,327 (30.5%) | 1.10 (1.05,1.16) | <0.001 | 1.09 (1.03,1.15) | 0.003 | 4,069 (37.3%) | 0.73 (0.70,0.77) | <0.001 | 0.74 (0.70,0.78) | <0.001 |
| 40+ yrs | 835 (33.7%) | 1.28 (1.17,1.40) | <0.001 | 1.19 (1.08,1.31) | <0.001 | 919 (37.1%) | 0.73 (0.67,0.79) | <0.001 | 0.72 (0.66,0.79) | <0.001 |
|  |  |  |  |  |  |  |  |  |  |  |
| **Gravidity** |  |  |  |  |  |  |  |  |  |  |
| 0 | 6,150 (31.4%) | 1.12 (1.07,1.18) | <0.001 | 1.07 (1.01,1.13) | 0.019 | 8,687 (44.4%) | 1.07 (1.02,1.12) | 0.006 | 0.93 (0.88,0.97) | 0.003 |
| 1 | 5,488 (28.2%) | 0.96 (0.92,1.01) | 0.143 | 0.97 (0.92,1.02) | 0.243 | 8,278 (42.5%) | 0.99 (0.94,1.03) | 0.611 | 0.94 (0.90,0.99) | 0.013 |
| 2 | 3,367 (28.9%) | 1.00 |  | 1.00 |  | 4,977 (42.8%) | 1.00 |  | 1.00 |  |
| ≥3 | 3,867 (31.6%) | 1.13 (1.07,1.20) | <0.001 | 1.07 (1.01,1.14) | 0.020 | 5,681 (46.4%) | 1.16 (1.10,1.22) | <0.001 | 1.14 (1.09,1.21) | <0.001 |
|  |  |  |  |  |  |  |  |  |  |  |
| **Child sex** |  |  |  |  |  |  |  |  |  |  |
| Male | 10,812 (33.5%) | 1.42 (1.37,1.46) | <0.001 | 1.59 (1.54,1.65) | <0.001 | 14,874 (46.1%) | 1.20 (1.17,1.24 | <0.001 | 1.22 (1.18,1.26) | <0.001 |
| Female | 8,067 (26.3%) | 1.00 |  | 1.00 |  | 12,752 (41.5%) | 1.00 |  | 1.00 |  |
|  |  |  |  |  |  |  |  |  |  |  |
| **Multiple birth** |  |  |  |  |  |  |  |  |  |  |
| Yes | 17,586 (28.7%) | 7.64 (6.83,8.54) | <0.001 | 3.74 (3.32,4.21) | <0.001 | 26,942 (44.0%) | 0.85 (0.77,0.94) | 0.001 | 0.82 (0.73,0.91) | <0.001 |
| No | 1,286 (75.5%) | 1.00 |  | 1.00 |  | 681 (40.0%) | 1.00 |  | 1.00 |  |
|  |  |  |  |  |  |  |  |  |  |  |
|  |  |  |  |  |  |  |  |  |  |  |
| **Prematurity** |  |  |  |  |  |  |  |  |  |  |
| <32wk | 657 (95.6%) | 63.85 (44.27,92.11) | <0.001 | 46.61 (34.32,71.73) | <0.001 | 373 (54.3%) | 1.56 (1.34,1.81) | <0.001 | 1.60 (1.37,1.88) | <0.001 |
| 32-36wk | 3,478 (75.8%) | 9.15 (8.53,9.82) | <0.001 | 7.80 (7.25,8.40) | <0.001 | 2,291 (50.0%) | 1.31 (1.23,1.39) | <0.001 | 1.36 (1.27,1.45) | <0.001 |
| >=37wk | 14,729 (25.5%) | 1.00 |  | 1.00 |  | 24,952 (43.3%) | 1.00 |  | 1.00 |  |
|  |  |  |  |  |  |  |  |  |  |  |
| **Birth weight** |  |  |  |  |  |  |  |  |  |  |
| Low birth weight (<2500g) | 3,138 (82.2%) | 12.69 (11.66,13.81) | <0.001 | 10.68 (9.77,11.67) | <0.001 | 1,903 (49.8%) | 1.29 (1.21,1.38) | <0.001 | 1.33 (1.24,1.43) | <0.001 |
| Normal birth weight (≥2500g) | 15,740 (26.6%) | 1.00 |  | 1.00 |  | 25,722 (43.5%) | 1.00 |  | 1.00 |  |
|  |  |  |  |  |  |  |  |  |  |  |
| **APGAR 5 score** |  |  |  |  |  |  |  |  |  |  |
| Lowest (least healthy) 1 | 38 (52.8%) | 3.00 (1.89,4.76) | <0.001 | 0.84 (0.49,1.42) | 0.509 | 19 (26.4%) | 0.47 (0.49,1.42) | 0.005 | 0.35 (0.20,0.61) | <0.001 |
| 2 | 124 (84.9%) | 15.11 (9.60,23.79) | <0.001 | 12.76 (7.90,20.61 | <0.001 | 76 (52.1%) | 1.42 (1.02,1.96) | 0.035 | 1.25 (0.89,1.75) | 0.197 |
| 3 | 547 (76.3%) | 8.63 (7.26,10.26) | <0.001 | 7.08 (5.90,8.50) | <0.001 | 378 (52.7%) | 1.46 (1.26,1.69) | <0.001 | 1.31 (1.13,1.53) | 0.001 |
| 4 | 2,384 (60.6%) | 4.12 (3.86,4.40) | <0.001 | 3.43 (3.20,3.68) | <0.001 | 1,964 (49.9%) | 1.30 (1.22,1.39) | <0.001 | 1.25 (1.17,1.33) | <0.001 |
| Highest (most healthy) 5 | 15,767 (27.2%) | 1.00 |  | 1.00 |  | 25,168 (43.4%) | 1.00 |  | 1.00 |  |

IRSD = Index of Relative Socio-Economic Disadvantage, ARIA = Accessibility/ Remoteness Index of Australia, OR = odds ratio, aOR = adjusted odds ratio, 95% CI = 95% confidence interval

*Adjusted for Indigenous status, IRSD (Index of Relative Socio-Economic Disadvantage), maternal age, gravidity, multiple birth, gender of child, birth weight

**Table C Risk of hospital admission in Indigenous and non-Indigenous infants aged 0-<1m (neonates) by socio economic status, 2010-2011**

|  | Total | | | | Indigenous | | | Non Indigenous | | |
| --- | --- | --- | --- | --- | --- | --- | --- | --- | --- | --- |
|  |  |  |  |  |  |  |  |  |  |  |
|  | Total children n=62,965 | Infants with at least one hospital admission n=11,977 | Unadjusted | Multivariable | Total Indigenous children | Indigenous infants with at least one hospital admission | Multivariable | Total non-Indigenous children | Non-Indigenous infants with at least one hospital admission | Multivariable |
|  |  |  | OR (95% CI) | OR (95% CI)* | n=3,382 | n=770 (22.8%)** | OR (95% CI)* | n=59,583 | n=11,207 (18.8%)** | OR (95% CI)* |
| Most disadvantaged 1 | 3,634 | 654 (18.0%) | 0.82 (0.75,0.90) | 0.84 (0.76,0.92) | 1,323 | 290 (21.9%) | 0.74 (0.56,0.96) | 2,311 | 364 (15.8%) | 0.79 (0.70,0.89) |
| 2 | 9,670 | 1850 (19.1%) | 0.88 (0.83,0.94) | 0.89 (0.84,0.95) | 406 | 99 (24.4%) | 0.92 (0.66,1.29) | 9,264 | 1751 (18.9%) | 0.89 (0.83,0.95) |
| 3 | 8,126 | 1248 (15.4%) | 0.68 (0.63,0.72) | 0.70 (0.65,0.75) | 473 | 93 (19.7%) | 0.71 (0.51,1.00) | 7,653 | 1155 (15.1%) | 0.69 (0.64,0.75) |
| 4 | 17,985 | 3252 (18.1%) | 0.82 (0.78,0.86) | 0.86 (0.82,0.91) | 600 | 140 (23.3%) | 0.86 (0.63,1.17) | 17,385 | 3112 (17.9%) | 0.86 (0.81,0.91) |
| Least disadvantaged 5 | 22,018 | 4658 (21.2%) | 1.00 | 1.00 | 478 | 121 (25.3%) | 1.00 | 21,540 | 4537 (21.1%) | 1.00 |
| Data missing | 1,532 | 315 (20.6%) | - | - | 102 | 27 (26.5%) | - | 1,430 | 288 (20.1%) | - |
| P value trend | - | - | <0.001 | <0.001 | - | - | 0.054 | - | - | <0.001 |

* Adjusted for maternal age, gravidity, multiple birth, gender of child, birth weight

OR = odds ratio, aOR = adjusted odds ratio, 95% CI = 95% confidence interval

**Risk of hospital admission in Indigenous compared to non-Indigenous infants aged 0-<1m: OR 1.27 (1.17, 1.38); aOR 1.25 (1.14, 1.38)

**Table D Risk of emergency department presentation in Indigenous and non-Indigenous infants aged 0-<1m (neonates) by socio economic status, 2010-2011**

|  | Total | | | | Indigenous | | | Non Indigenous | | |
| --- | --- | --- | --- | --- | --- | --- | --- | --- | --- | --- |
|  |  |  |  |  |  |  |  |  |  |  |
|  | Total children n=62,965 | Infants with at least one emergency department presentation n=2,818 | Unadjusted | Multivariable | Total Indigenous children | Indigenous infants with at least one emergency department presentation | Multivariable | Total non-Indigenous children | Non-Indigenous infants with at least one emergency department presentation n=2,543 (4.3%)** | Multivariable |
|  |  |  | OR (95% CI) | OR (95% CI)* | n=3,382 | n=275 (8.1%)** | OR (95% CI)* | n=59,583 |  | OR (95% CI)* |
| Most disadvantaged 1 | 3,634 | 245 (6.7%) | 1.77 (1.53,2.05) | 1.51 (1.30,1.75) | 1,323 | 125 (9.5%) | 1.15 (0.79,1.67) | 2,311 | 120 (5.2%) | 1.24 (1.02,1.51) |
| 2 | 9,670 | 488 (5.1%) | 1.30 (1.16,1.46) | 1.20 (1.07,1.35) | 406 | 29 (7.1%) | 0.85 (0.52,1.40) | 9,264 | 459 (5.0%) | 1.22 (1.08,1.37) |
| 3 | 8,126 | 403 (5.0%) | 1.28 (1.13,1.44) | 1.16 (1.03,1.31) | 473 | 40 (8.5%) | 1.00 (0.63,1.59) | 7,653 | 363 (4.7%) | 1.15 (1.01,1.31) |
| 4 | 17,985 | 756 (4.2%) | 1.07 (0.97,1.19) | 0.99 (0.90,1.10) | 600 | 32 (5.3%) | 0.60 (0.37,0.98) | 17,385 | 724 (4.2%) | 1.01 (0.91,1.12) |
| Least disadvantaged 5 | 22,018 | 865 (3.9%) | 1.00 | 1.00 | 478 | 40 (8.4%) | 1.00 | 21,540 | 825 (3.8%) | 1.00 |
| Data missing | 1,532 | 61 (4.0%) | - | - | 102 | 9 (8.8%) | - | 1,430 | 52 (3.6%) | - |
| P value trend | - | - | <0.001 | <0.001 | - | - | 0.044 | - | - | <0.001 |

* Adjusted for maternal age, gravidity, multiple birth, gender of child, birth weight

OR = odds ratio, aOR = adjusted odds ratio, 95% CI = 95% confidence interval

**Risk of emergency department presentation in Indigenous compared to non-Indigenous infants aged 0-<1m: OR 1.99 (1.74, 2.26); aOR 1.52 (1.32, 1.75)

**Table E Risk of hospital admission in Indigenous and non-Indigenous infants aged 1-11m (post neonates) by socio economic status, 2010-2011**

|  | Total | | | | Indigenous | | | Non Indigenous | | |
| --- | --- | --- | --- | --- | --- | --- | --- | --- | --- | --- |
|  |  |  |  |  |  |  |  |  |  |  |
|  | Total children n=62,965 | Infants with at least one hospital admission n=9,993 | Unadjusted | Multivariable | Total Indigenous children | Indigenous infants with at least one hospital admission | Multivariable | Total non-Indigenous children | Non-Indigenous infants with at least one hospital admission | Multivariable |
|  |  |  | OR (95% CI) | OR (95% CI)* | n=3,382 | n=1,061 (31.4%)** | OR (95% CI)* | n=59,583 | n=8,932 (15.0%)** | OR (95% CI)* |
| Most disadvantaged 1 | 3,634 | 889 (24.5%) | 1.96 (1.80,2.13) | 1.67 (1.53,1.82) | 1,323 | 479 (36.2%) | 1.76 (1.38,2.25) | 2,311 | 410 (17.7%) | 1.28 (1.14,1.44) |
| 2 | 9,670 | 1493 (15.4%) | 1.10 (1.03,1.18) | 1.03 (0.96,1.10) | 406 | 113 (27.8%) | 1.21 (0.89,1.65) | 9,264 | 1380 (14.9%) | 1.02 (0.95,1.10) |
| 3 | 8,126 | 1353 (16.7%) | 1.21 (1.13,1.30) | 1.14 (1.06,1.22) | 473 | 138 (29.2%) | 1.33 (0.99,1.79) | 7,653 | 1215 (15.9%) | 1.12 (1.04,1.20) |
| 4 | 17,985 | 2879 (16.0%) | 1.15 (1.09,1.22) | 1.10 (1.04,1.16) | 600 | 184 (30.7%) | 1.37 (1.04,1.82) | 17,385 | 2695 (15.5%) | 1.09 (1.03,1.15) |
| Least disadvantaged 5 | 22,018 | 3123 (14.2%) | 1.00 | 1.00 | 478 | 115 (24.1%) | 1.00 | 21,540 | 3008 (14.0%) | 1.00 |
| Data missing | 1,532 | 256 (16.7%) | - | - | 102 | 32 (31.4%) | - | 1,430 | 224 (15.7%) | - |
| P value trend | - | - | <0.001 | <0.001 | - | - | <0.001 | - | - | 0.005 |

* Adjusted for maternal age, gravidity, multiple birth, gender of child, birth weight

OR = odds ratio, aOR = adjusted odds ratio, 95% CI = 95% confidence interval

**Risk of hospital admission in Indigenous compared to non-Indigenous infants aged 1-11m: OR 2.59 (2.40, 2.80); aOR 1.87 (1.72, 2.03)

**TableF Risk of emergency department presentation in Indigenous and non-Indigenous infants aged 1-11m (post neonates) by socio economic status. , 2010-2011**

|  | Total | | | | Indigenous | | | Non Indigenous | | |
| --- | --- | --- | --- | --- | --- | --- | --- | --- | --- | --- |
|  |  |  |  |  |  |  |  |  |  |  |
|  | Total children n=62,965 | Infants with at least one emergency department presentation n=26,494 | Unadjusted | Multivariable | Total Indigenous children | Indigenous infants with at least one emergency department presentation | Multivariable | Total non-Indigenous children | Non-Indigenous infants with at least one emergency department presentation n=24,149 (40.5%)** | Multivariable |
|  |  |  | OR (95% CI) | OR (95% CI)* | n=3,382 | n=2,345 (69.3%)** | OR (95% CI)* | n=59,583 |  | OR (95% CI)* |
| Most disadvantaged 1 | 3,634 | 2359 (64.9%) | 3.21 (2.98,3.45) | 2.67 (2.48,2.88) | 1,323 | 967 (73.1%) | 1.38 (1.10,1.73) | 2,311 | 1392 (60.2%) | 2.47 (2.26,2.70) |
| 2 | 9,670 | 4230 (43.7%) | 1.35 (1.28,1.42) | 1.23 (1.17,1.29) | 406 | 265 (65.3%) | 0.96 (0.72,1.27) | 9,264 | 3965 (42.8%) | 1.23 (1.17,1.29) |
| 3 | 8,126 | 3804 (46.8%) | 1.53 (1.45,1.61) | 1.38 (1.31,1.46) | 473 | 331 (70.0%) | 1.20 (0.91,1.58) | 7,653 | 3473 (45.4%) | 1.36 (1.29,1.44) |
| 4 | 17,985 | 7393 (41.1%) | 1.21 (1.16,1.26) | 1.11 (1.06,1.16) | 600 | 395 (65.8%) | 0.96 (0.74,1.24) | 17,385 | 6998 (40.3%) | 1.11 (1.07,1.16) |
| Least disadvantaged 5 | 22,018 | 8053 (36.6%) | 1.00 | 1.00 | 478 | 315 (65.9%) | 1.00 | 21,540 | 7738 (35.2%) | 1.00 |
| Data missing | 1,532 | 655 (42.8%) | - | - | 102 | 72 (70.6%) | - | 1,430 | 583 (40.8%) | - |
| P value trend | - | - | <0.001 | <0.001 | - | - | 0.001 | - | - | <0.001 |

* Adjusted for maternal age, gravidity, multiple birth, gender of child, birth weight

OR = odds ratio, aOR = adjusted odds ratio, 95% CI = 95% confidence interval

**Risk of emergency department presentation in Indigenous compared to non-Indigenous infants aged 1-11m: OR 3.32 (3.08, 3.58); aOR 2.17 (2.00, 2.35)
